# Synthesis and In Silico Study of Some New *bis*-[1,3,4]thiadiazolimines and *bis*-Thiazolimines as Potential Inhibitors for SARS-CoV-2 Main Protease

**DOI:** 10.3390/cimb44100311

**Published:** 2022-09-30

**Authors:** Sobhi M. Gomha, Sayed M. Riyadh, Magda H. Abdellattif, Tariq Z. Abolibda, Hassan M. Abdel-aziz, AbdElAziz. A. Nayl, Alaa M. Elgohary, Abdo A. Elfiky

**Affiliations:** 1 Department of Chemistry, Faculty of Science, Islamic University of Madinah, Madinah 42351, Saudi Arabia; 2 Department of Chemistry, Faculty of Science, Cairo University, Giza 12613, Egypt; 3 Department of Chemistry, Faculty of Science, Taibah University, Al-Madinah Al-Munawarah 30002, Saudi Arabia; 4 Department of Chemistry, College of Science, Taif University, P.O. Box 11099, Taif 21944, Saudi Arabia; 5 Department of Chemistry, Faculty of Science, Bani Suef University, Bani Suef 62521, Egypt; 6 Department of Chemistry, College of Science, Jouf University, Sakaka 72341, Saudi Arabia; 7 Department of Biophysics, Faculty of Science, Cairo University, Giza 12613, Egypt

**Keywords:** *bis*-(3-phenylthiourea), hydrazonoyl halides, *α*-haloketones, molecular docking, SARS-CoV-2 M^pro^

## Abstract

A novel series of *bis*-[1,3,4]thiadiazolimines, and *bis*-thiazolimines, with alkyl linker, were synthesized through general routes from cyclization of 1,1′-(hexane-1,6-diyl)bis(3-phenylthiourea) and hydrazonoyl halides or *α*-haloketones, respectively. Docking studies were applied to test the binding affinity of the synthesized products against the M^pro^ of SARS-CoV-2. The best compound, **5h**, has average binding energy (−7.50 ± 0.58 kcal/mol) better than that of the positive controls O6K and N3 (−7.36 ± 0.34 and −6.36 ± 0.31 kcal/mol). Additionally, the docking poses (H-bonds and hydrophobic contacts) of the tested compounds against the M^pro^ using the PLIP web server were analyzed.

## 1. Introduction

Recently, COVID-19 vaccines have been developed and evaluated to be validated for use [1,2,3,4]. Thus, many researchers orchestrated their efforts to formulate and construct novel bioactive *bis*-heterocycles as antiviral agents and other therapeutic effects. It has been reported in the literature that compounds containing *bis*-thiadiazole cores have attracted considerable interest in the area of drug discovery due to their potential as antihypertensive *α*-blocking [5], antimicrobial [6,7,8], and anticancer [9,10] agents. In addition, *bis*-thiazole pharmacophores were reported to reveal various biological activities such as anti-inflammatory, analgesic, anti-ulcerogenic [11], antiviral [12], antimicrobial [13,14], antioxidant [15], and anticancer activities [16,17,18,19]. Moreover, azoles tethered imines are accentuated to investigate new potential drug candidates with diverse therapeutic efficacy such as antibacterial, antimicrobial, antifungal, anticancer, antioxidant, and antiproliferative activities [20,21,22,23,24,25]. So, molecular hybridization of imines and azoles is a beneficial approach to structural alteration, as a single species of two or more pharmacophores can serve as potential COVID-19 drug candidates [26]. 

*bis*-1,3-Thiazole derivatives were examined against different viruses (such as hepatitis B and C viruses, poliovirus, and influenza A virus) and showed promising results (up to EC50 0.56 μM) [12].

The main protease (M^pro^) or 3CL^Pro^ is a vital viral protein that is important for the SARS-CoV-2 life cycle. Its function is to process viral polyprotein upon entry and replication in the host cell [27]. Due to its conservation among different coronaviruses, it is one of the most studied targets in SARS-CoV-2 research, while the dimer form represents the active conformation of the functional enzyme [28,29,30,31]. Some drugs are potential candidates that block M^pro^ function, such as Paxlovid, which consists of two drugs, nirmatrelvir (protease inhibitor) and ritonavir (nirmatrelvir bioavailability enhancer). Paxlovid was approved for COVID-19 patients in Europe, while other drugs are in clinical trials [32,33,34].

In this study, the combination of *bis*-thiadiazoles or *bis*-thiazoles with imine moiety in a hybridized molecule was achieved, and the synthesized derivatives were docked against the main protease (M^pro^) of SARS-CoV-2. The docked complexes are analyzed using molecular modeling tools, including molecular docking and dynamics simulation.

## 2. Results and Discussion

**Scheme 1:**
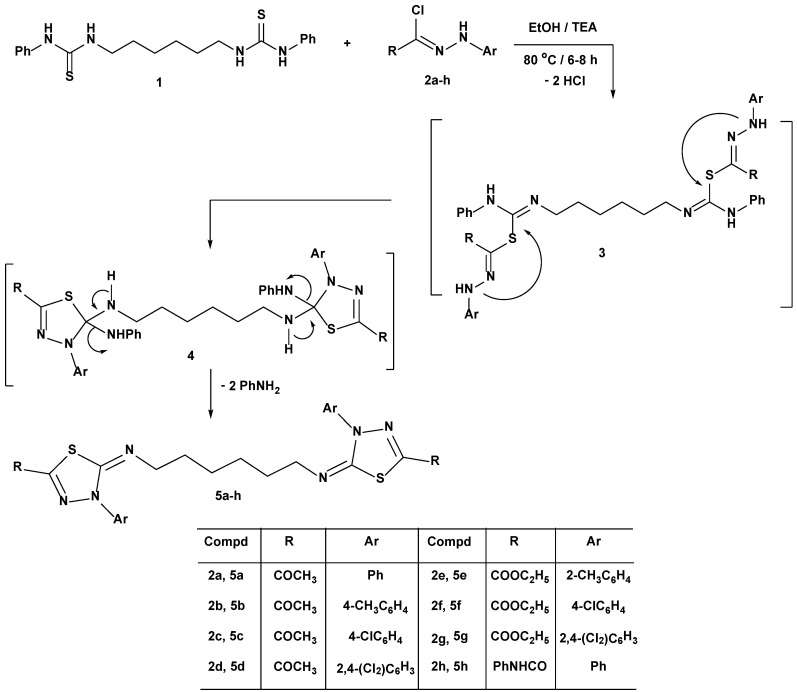
Synthesis of *bis*-[1,3,4]thiadiazolimines **5a**–**h**.

1,1′-(Hexane-1,6-diyl)bis(3-phenylthiourea) (**1**), derived from hexamethylenediamine and phenyl isothiocyanate [35,36], was allowed to react with hydrazonoyl chlorides **2** in ethanolic solution containing a few drops of triethylamine resulting in the formation of *N*,*N*′-(hexane-1,6-diyl)bis[5-substituted-3-aryl-1,3,4-thiadiazol-2(3*H*)-imine] (**5a**–**h**) as target compounds. The synthetic pathway is depicted in Scheme 1. 

The synthesis of *bis*-[1,3,4]thiadiazol-2-imines **5a**–**h** was inaugurated by nucleophilic halogen displacement by the thiol group to afford intermediate **3**. Intramolecular cyclization of the latter intermediate with consecutive elimination of aniline molecules furnished the isolated products in good yields (Scheme 1). The spectroscopic data (IR, ^1^H-NMR, ^13^C-NMR, and MS) and the elemental analyses of *bis*-[1,3,4]thiadiazol-2-imines **5a**–**h** were in agreement with the assigned structures (see Experimental section).

**Scheme 2:**
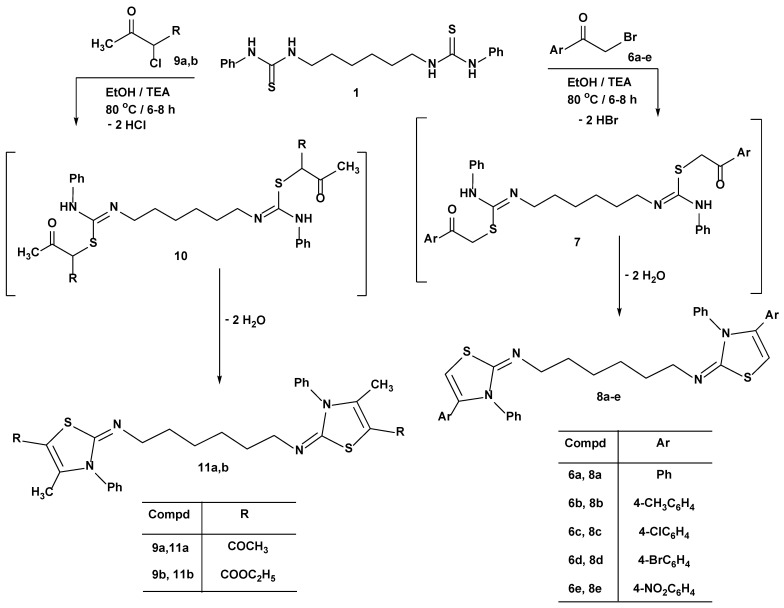
Synthesis of *bis*-thiazolimies **8a**–**e** and **11a**,**b**.

The scope of the previous hetero-cyclization was expanded with respect to *α*-haloketones. Thus, we explored the cyclo-condensation reaction of 1,1′-(hexane-1,6-diyl)bis(3-phenylthiourea) (**1**) with substituted phenacyl bromides **6a**–**e** or 3-chloro-2,4-pentanedione (**9a**) or ethyl 2-chloro-3-oxobutanoate (**9b**) under the employed conditions and the desired *bis*-thiazol-2-imines **8a**–**e**, or **11a**, or **11b**, respectively, were obtained in acceptable yields through the proposed mechanistic pathway illustrated in Scheme 2. This indicated that the synthetic utility of *bis*(3-phenylthiourea) with an alkyl linker could improve *bis*-heterocyclic scaffolds, enhancing their biological activities. 

## 3. Computational Analysis

Molecular docking combined with molecular dynamics simulation was utilized to test the binding affinity of starting material **1**, *bis*-[1,3,4]thiadiazol-2-imines **5a**–**h**, and *bis*-thiazol-2-imines (**8a**–**e** and **11a**,**b**) against the M^pro^ of SARS-CoV-2. The solved structure used in this study is the dimeric M^pro^ (PDB ID: 6Y2G), this is due to the fact that it is the active dimeric form of the M^pro^ and solved with O6K inhibitor (tert-butyl (1-((S)-1-(((S)-4-(benzylamino)-3,4-dioxo-1-((S)-2-oxopyrrolidin-3-yl)butan-2-yl)amino)-3-cyclopropyl-1-oxopropan-2-yl)-2-oxo-1,2-dihydropyridin-3-yl)carbamate (alpha-ketoamide 13b)), so we extracted it and used it as positive control. Additionally, we used the other inhibitor N3 (N-[(5-methylisoxazol-3-yl)carbonyl]alanyl-L-valyl-N~1~-((1R,2Z)-4-(benzyloxy)-4-oxo-1-{[(3R)-2-oxopyrrolidin-3-yl]methyl}but-2-enyl)-L-leucinamide) found in the solved structure 6LU7 to compare its affinity to M^pro^ to that of *bis*-[1,3,4]thiadiazolimines and *bis*-thiazolimines against the active site of the M^pro^.

**Figure 1:**
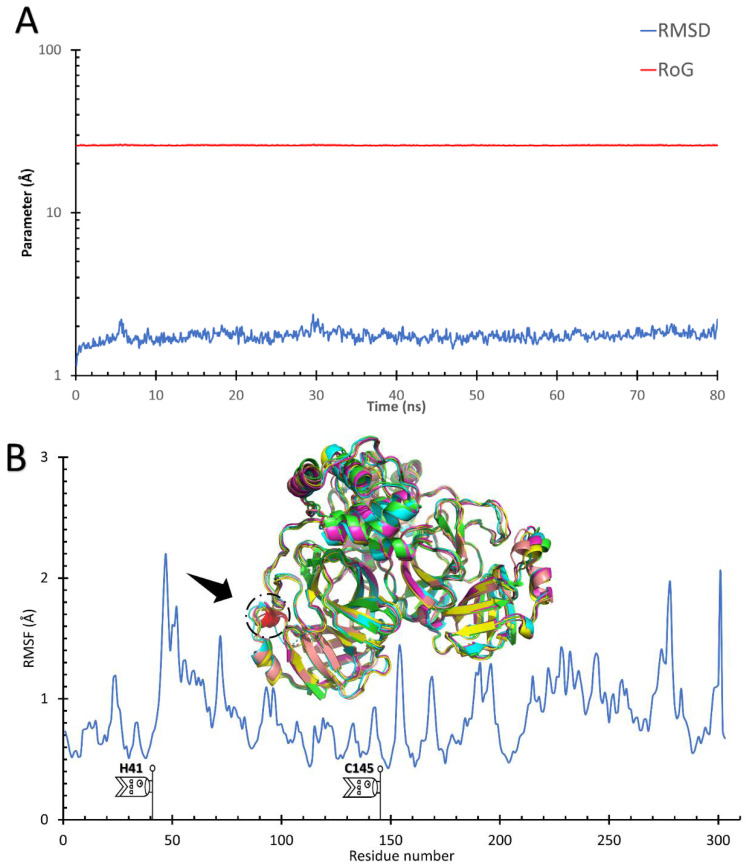
Molecular dynamics simulation analysis. (**A**) The root-mean-square deviation (RMSD) (blue) and the radius of gyration (RoG) (red) versus the simulation time. (**B**) The per-residue root-mean-square fluctuations (RMSF) and the five representative structures of M^pro^. Active site residues (H41 and C145) are marked on the RMSF curve and the structures.

The docking was performed after equilibrating the structure for an 80 ns MDS run. The reason for performing MDS before docking is that we remove ligands and want the protein to be relaxed before making the docking calculations. We also equilibrate the system and cluster the trajectories to test different possible protein conformations against the compounds. Figure 1A reflects the system equilibration as the root-mean-square deviation (RMSD) curve (blue) is flattened around 2 Å. Additionally, the radius of the gyration (RoG) curve (red) is flat during the simulation period, averaging about 26 Å. Figure 1B shows the five representative models for M^pro^ after clustering the trajectories depicted in colored cartoons. Additionally, the per-residue root-mean-square fluctuations (RMSF) in Å are plotted. The active site residues (H41 and C145) are marked on the RMSF curve. In addition to the protein termini, only one region (S46-M49) shows moderate fluctuations (RMSF < 1.5 Å). This region is colored in red in the structures. The active site residues are away from this region and show low fluctuations (RMSF > 0.7 Å). 

**Figure 2:**
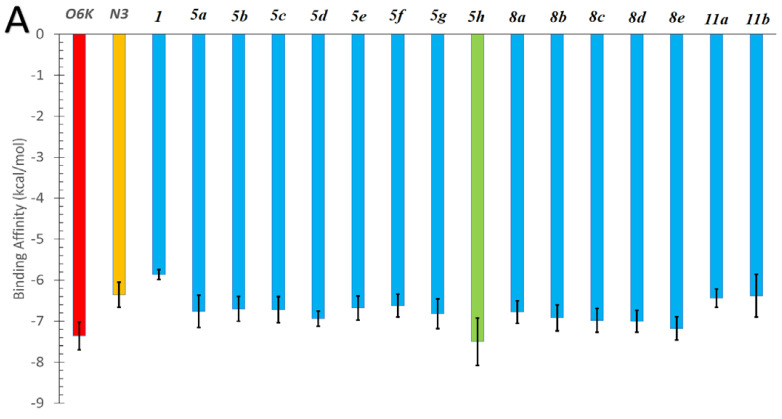

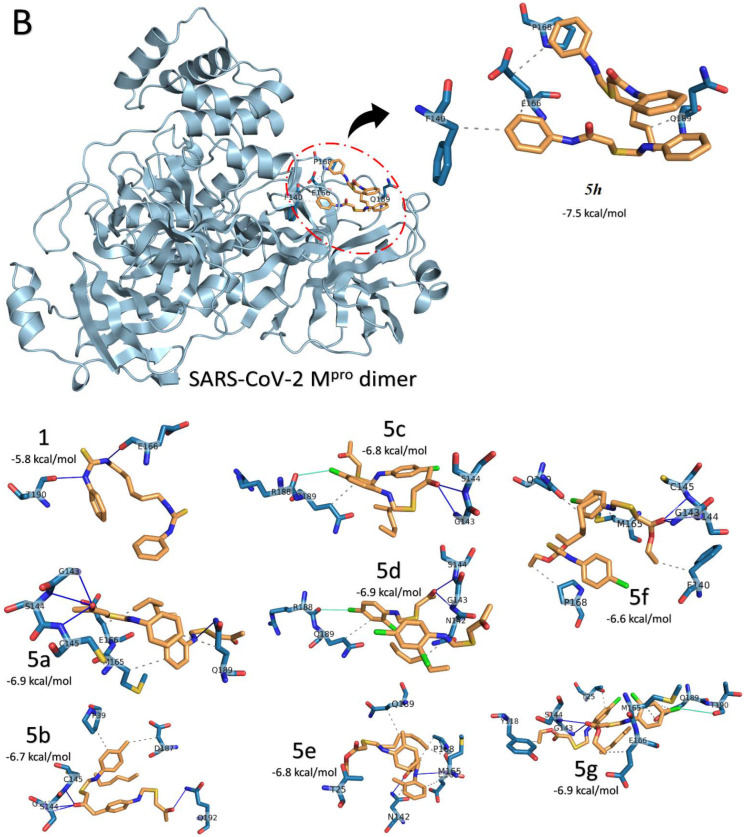

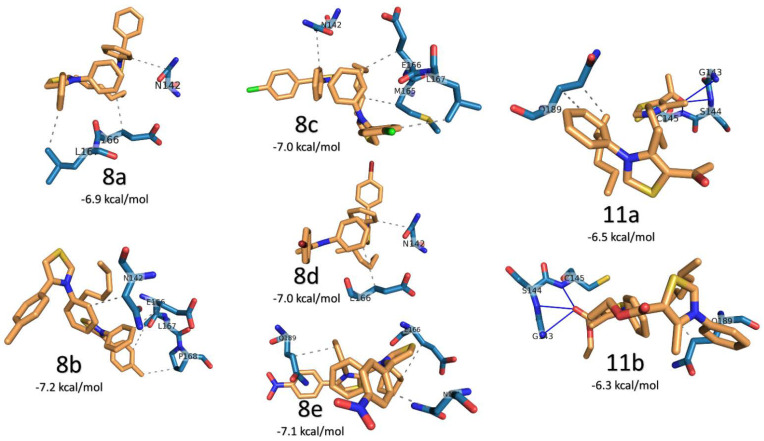
(**A**) Average binding energies (in kcal/mol) of the positive controls [O6K(red) and N3 (orange)] and compounds **1**, **5a**–**h**, **8a**–**e**, and **11a**,**b** (blue) docked into the active site residues of SARS-CoV-2 M^pro^. Error bars represent the standard deviations. The best compound in binding the SARS-CoV-2 M^pro^ (**5h**) is shown in green. (**B**) The docking complexes of compounds against SARS-CoV-2 M^pro^ are depicted by PyMOL software.

The bar graph of Figure 2A shows the average (out of five values) binding energy (in kcal/mol) against the M^pro^ of different SARS-CoV-2 conformations after MDS trajectory clustering. The error bars represent the standard deviation which was calculated by the Microsoft Excel formula $\sqrt{\frac{\sum {\left(x-\bar{x}\right)}^{2}}{n-1}}$. Before the docking of the compounds, we tested the docking system by performing a redocking of O6K to the solved structure and we obtained a root-mean-square deviation of 0.92 Å (1806 fitted atoms). The positive controls O6K and N3 (putative inhibitors to M^pro^ found in the solved structures 6Y2G and 6LU7) are shown in red and orange, respectively, while the compounds **1**, **5a**–**h**, **8a**–**e**, and **11a**,**b** are in blue (Figure 2A). The best compound, **5h**, is shown in green. The compound **5h** has average binding energy (−7.50 ± 0.58 kcal/mol) better than that of the positive controls O6K and N3 (−7.36 ± 0.34 and −6.36 ± 0.31 kcal/mol). Despite the fact that the binding affinity value of **5h** compound is not significantly different compared to the positive control, it is still a potential anti-SARS-CoV-2 M^pro^ compound that deserves further investigation. 

Figure 2B shows the detailed interaction pattern between **5h** and the M^pro^. Five hydrophobic contacts are formed between **5h** and the residues F140, E166(2), P168, and Q189 of M^pro^. Additionally, the other tested compounds (except compound **1**) present enhanced binding energies against M^pro^ (−6.38 ± 0.52 down to −7.18 ± 0.29 kcal/mol for **11b** and **8e**, respectively) compared to the positive control N3. This reflects their potential to be tightly bound to and might inhibit the M^pro^ of SARS-CoV-2.

Furthermore, we analyzed the docking poses of the compounds against the M^pro^ using the PLIP web server, and a detailed list is tabulated (Table 1). The ligand that has the nearest binding affinity to the average value is represented. These modes resemble five different protein conformations after an 80 ns MDS run. For the compounds **5a**, **5b**, **5c**, **5d**, **5e**, **5f**, **5g**, **11a**, and **11b,** the most common interactions are the H-bonds (three) and the hydrophobic contacts (three). On the other hand, only hydrophobic contacts (four) are reported in compounds **5h**, **8a**, **8b**, **8c**, **8d**, and **8e**, while compound **1** formed only two H-bonds with the M^pro^. Compounds **5c**, **5d**, and **5g** form a halogen bond with R188 or T190 residues of the M^pro^. The highest contributing residues in the formed interactions between the compounds and the Mpro are Q189, E166, N142, G143, S144, and C145, which formed 14, 11, 8, 8, 8, and 5 interactions, respectively. 

**Figure 3:**
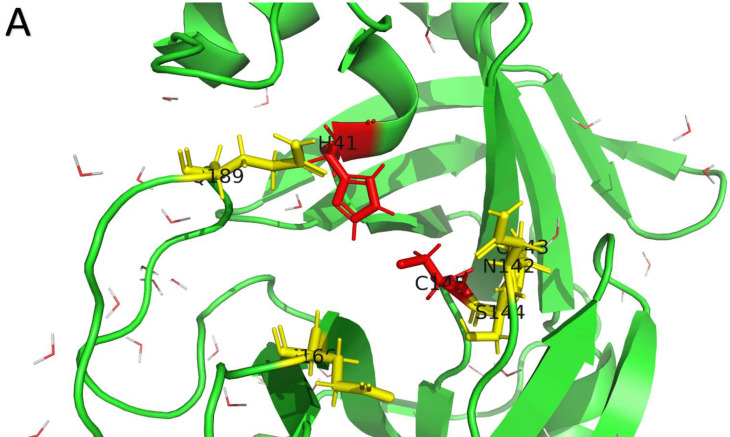

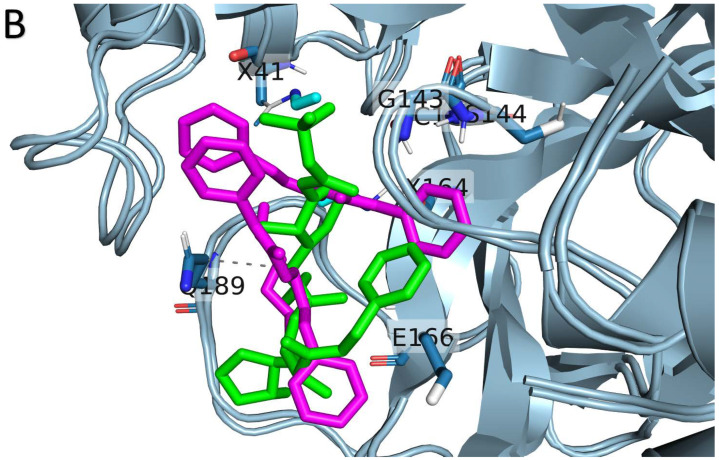
(**A**) The most reported interacting residues of SARS-CoV-2 Mpro (green cartoon) with the ligands are represented using the solved structure (PDB ID: 6LU7). The active site dyads (H41 and C145) are represented by red sticks, while the yellow sticks represent the most reported interacting residues, N142, G143, S144, E166, and Q189 (underlined in Table 1). (**B**) The superposition of the M^pro^ (blue cartoon) is complexed with O6K (green sticks) and 5h (magenta sticks) for comparison. The active site pocket residues are labelled with its 1-letter codes.

The binding affinities are listed among the number of H-bonds, halogen bonds, and hydrophobic contacts and the residues that take part in their formation in Table 1. Bold residues are the common residues interacting with the ligands, while the active site dyads are underlined. These residues are essential in the protease function as they lie within the active site pocket. When some small molecules block this pocket, it may interfere with the protease function (Figure 3A), which is yet to be verified experimentally. The best compound 5h (magenta sticks) is fitted in the active site pocket similar to the positive control O6K (green sticks) (Figure 3B). This indicates its possible usefulness as a SARS-CoV-2 M^pro^ inhibitor. According to the SwissADME web tool (http://www.swissadme.ch/index.php) (accessed on 6 June 2016), compound **5h** has poor solubility, has low gastrointestinal absorption, has a 674.84 g/mol molecular weight, 0.17 bioavailability score, and does not have adverse pharmacokinetics properties.

**Figure 4:**
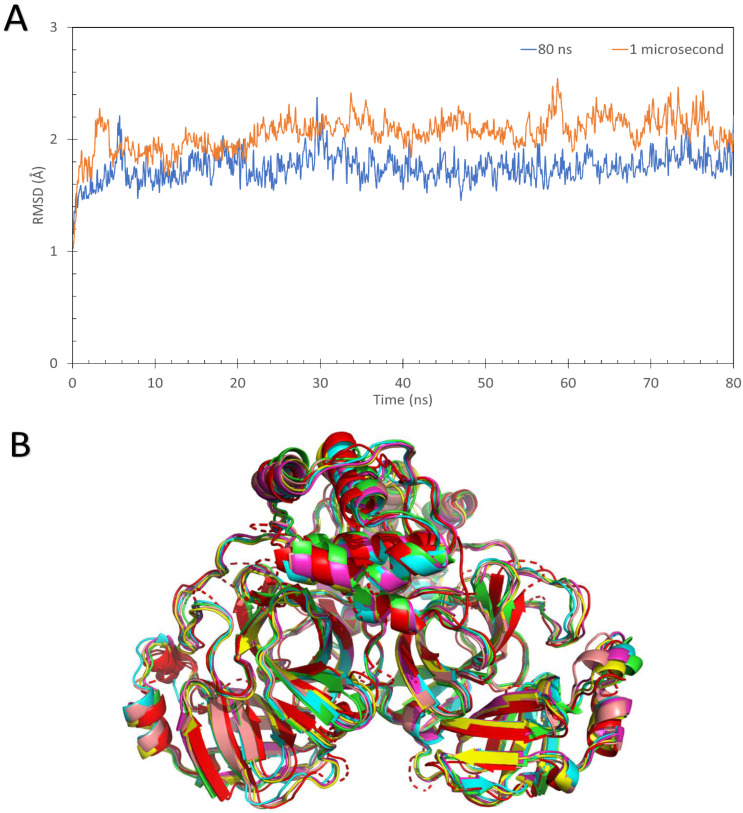

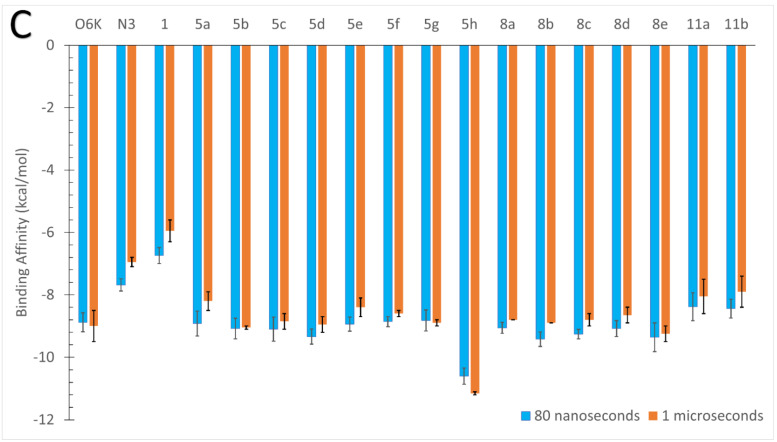
**Comparison between the 80 ns and the 1 microsecond trajectories**. (**A**) The root-mean-square deviation versus the simulation time. (**B**) The superposition of the most abundant conformation over the 1 microsecond trajectory (red cartoon) with the five different conformations of the 80 ns trajectory (other colored cartoons). (**C**) The average binding energies were calculated using AutoDock Vina over PyRx software (blind) for both systems. Error bars represent the standard deviation.

We trust our simulation as the RMSD of our trajectory coincides with that of the large simulation trajectory found at the following link: (https://figshare.com/search?q=10.6084%2Fm9.figshare.12009789) (accessed on 23 March 2020). Figure 4A shows the RMSD of our simulation trajectory (80 ns) in blue and the 1 microsecond trajectory in red. Additionally, we performed blind docking of the compounds utilizing AutoDock Vina implemented in PyRx software against both systems (after trajectory clustering) and found comparable results. The compounds show comparable results to positive controls, and **5h** was the best based on the average binding affinities in both systems.

### 3.1. Experimental

Melting points were measured on an Electrothermal IA 9000 series digital melting point apparatus. IR spectra were recorded on Pye Unicam SP 3300 and Shimadzu FTIR 8101 PC infrared spectrophotometers. NMR spectra were recorded on a Varian Mercury VX-300 NMR spectrometer operating at 300 MHz (^1^H-NMR) and 75 MHz (^13^C-NMR) and run in deuterated dimethylsulfoxide (DMSO-*d*_6_). Chemical shifts were related to that of the solvent. Mass spectra were recorded on a Shimadzu GCeMS-QP1000 EX mass spectrometer at 70 eV. Elemental analyses were measured by using a German made Elementar vario LIII CHNS analyzer.

**Synthesis of 1,1′-(hexane-1,6-diyl)bis(3-phenylthiourea) (1).**

Phenyl isothiocyanate (20 mmol) was added dropwise to a stirred solution of 1,6-diaminohexane (10 mmol) in 30 mL of DMF. The mixture was stirred at room temperature for 3 h and then added to ice/water mixture. A precipitate was formed and was recrystallized from ethanol as colorless crystals.

Yield (96%); m.p. 149–151° (Lit. m.p. 148–149° [35,36]); IR: *v* 3344, 3222 (NH), 1560 (C=N), 1366 (C=S), 976 (C-N) cm^−1^; ^1^H-NMR *δ* = 1.24 (m, 4H, 2CH_2_), 1.46 (m, 4H, 2CH_2_), 3.38–3.41 (m, 4H, 2CH_2_), 7.16–7.42 (m, 10H, Ar-H), 7.62 (br, s, 2H, 2NH), 9.44 (br s, 2H, 2NHPh) ppm; MS *m*/*z* (%): 386 (M^+^, 47). Anal. Calcd. for C_20_H_26_N_4_S_2_ (386.16): C, 62.14; H, 6.78; N, 14.49; S, 16.69. Found C, 62.04; H, 6.59; N, 14.33; S, 16.55%.

**Synthesis of bis[1,3,4]thiadiazolimines and *bis*-thiazolimines.**

A mixture of 1,1′-(hexane-1,6-diyl)bis(3-phenylthiourea) (**1**) (0.386 g, 1 mmol) and appropriate hydrazonoyl halides **2a**–**h** or *α*-haloketones **6a**–**e** or **9a**,**b** (2 mmol of each) in ethanol (20 mL) containing triethylamine (0.1 g, 1 mmol) was refluxed for 6–8 h. (monitored by TLC). The formed precipitate was isolated by filtration, washed with methanol, dried, and recrystallized from EtOH to give products **5a**–**h** or **8a**–**e** or **11a**,**b**, respectively.

**1,1′-[Hexane-1,6-diyl-bis(azaneylylidene)]bis[(4-phenyl-4,5-dihydro-1,3,4-thiadiazol-2-yl-5-ylidene)]bis(ethan-1-one) (5a).**

Yellow crystals (70%); m.p. 173–175 °C; IR: *v* 3024, 2932 (C-H), 1691 (C=O), 1623 (C=N) cm^−1^; ^1^H-NMR (DMSO-*d*_6_): *δ* (ppm) 1.04–1.06 (m, 4H, -CH_2_CH_2_CH_2_CH_2_CH_2_CH_2_-), 1.16–1.21 (m, 4H, -CH_2_CH_2_CH_2_ CH_2_CH_2_CH_2_-), 2.44 (s, 6H, 2CH_3_), 3.37–3.42 (m, 4H, -CH_2_CH_2_CH_2_CH_2_CH_2_CH_2_-), 6.97–7.60 (m, 10H, Ar-H); ^13^C-NMR (DMSO-*d*_6_): *δ* (ppm) 21.12 (CH_2_), 24.36 (CH_3_), 26.81 (CH_2_), 46.51 (CH_2_-N), 124.51, 127.62, 129.21, 133.15, 145.61, 153.11 (Ar-C), 181.42 (C=O); MS *m*/*z* (%): 520 (M^+^, 71). Anal. Calcd. for C_26_H_28_N_6_O_2_S_2_ (520.17): C, 59.98; H, 5.42; N, 16.14; S, 12.31. Found C, 59.80; H, 5.31; N, 16.07; S, 12.19%.

**1,1′-[Hexane-1,6-diyl-bis(azaneylylidene)]bis[4-(p-tolyl)-4,5-dihydro-1,3,4-thiadiazole-2-yl-5-ylidene)]bis(ethan-1-one) (5b).**

Yellow crystals (75%); m.p. 161–163 °C; IR: *v* 3025, 2924 (C-H), 1707 (C=O), 1616 (C=N) cm^−1^; ^1^H-NMR (DMSO-*d*_6_): *δ* 1.03–1.05 (m, 4H, -CH_2_CH_2_CH_2_CH_2_CH_2_CH_2_-), 1.19–1.52 (m, 4H, -CH_2_CH_2_CH_2_ CH_2_CH_2_CH_2_-), 2.33 (s, 6H, 2Ar-CH_3_), 2.44 (s, 6H, 2CH_3_), 3.37–3.41 (m, 4H, -CH_2_CH_2_CH_2_CH_2_CH_2_CH_2_-), 6.97–7.54 (m, 8H, Ar-H); ^13^C-NMR (DMSO-*d*_6_): *δ* (ppm) 21.19 (CH_2_), 23.41 (CH_3_), 24.28 (CH_3_), 26.83 (CH_2_), 46.72 (CH_2_-N), 124.51, 127.55, 129.74, 132.95, 145.84, 153.17 (Ar-C), 180.81 (C=O); MS *m*/*z* (%): 548 (M^+^, 46). Anal. Calcd. for C_28_H_32_N_6_O_2_S_2_ (548.20): C, 61.29; H, 5.88; N, 15.32; S, 11.69. Found C, 61.16; H, 5.93; N, 15.18; S, 11.75%.

**1,1′-[Hexane-1,6-diyl-bis(azaneylylidene)]bis[4-(4-chlorophenyl)-4,5-dihydro-1,3,4-thiadiazole-2-yl-5-ylidene)]bis(ethan-1-one) (5c).**

Yellow crystals (77%); m.p. 180–182 °C; IR: *v* 3022, 2931 (C-H), 1701 (C=O), 1619 (C=N) cm^−1^; ^1^H-NMR (DMSO-*d*_6_): *δ* 1.17–1.21 (m, 4H, -CH_2_CH_2_CH_2_CH_2_CH_2_CH_2_-), 1.51–1.59 (m, 4H, -CH_2_CH_2_CH_2_ CH_2_CH_2_CH_2_-), 2.49 (s, 6H, 2CH_3_), 3.45–3.47 (m, 4H, -CH_2_CH_2_CH_2_CH_2_CH_2_CH_2_-), 6.97–7.64 (m, 8H, Ar-H); ^13^C-NMR (DMSO-*d*_6_): *δ* (ppm) 21.18 (CH_2_), 24.34 (CH_3_), 26.78 (CH_2_), 45.81 (CH_2_-N), 122.91, 125.62, 129.13, 134.11, 146.61, 153.18 (Ar-C), 183.41 (C=O); MS *m*/*z* (%): 590 (M^+^ +2, 11), 588 (M^+^, 38). Anal. Calcd. for C_26_H_26_Cl_2_N_6_O_2_S_2_ (588.09): C, 52.97; H, 4.45; N, 14.26; S, 10.88. Found C, 52.83; H, 4.41; N, 14.11; S, 11.02%.

**1,1′-[Hexane-1,6-diyl-bis(azaneylylidene)]bis[4-(2,4-dichlorophenyl)-4,5-dihydro-1,3,4-thiadiazole-2-yl-5-ylidene)]bis(ethan-1-one) (5d).**

Yellow crystals (77%); m.p. 179–181 °C; IR: *v* 3028, 2921 (C-H), 1704 (C=O), 1617 (C=N) cm^−1^; ^1^H-NMR (DMSO-*d*_6_): *δ* 1.17–1.22 (m, 4H, -CH_2_CH_2_CH_2_CH_2_CH_2_CH_2_-), 1.51–1.58 (m, 4H, -CH_2_CH_2_CH_2_ CH_2_CH_2_CH_2_-), 2.43 (s, 6H, 2CH_3_), 3.41–3.47 (m, 4H, -CH_2_CH_2_CH_2_CH_2_CH_2_CH_2_-), 6.96-7.68 (m, 6H, Ar-H); ^13^C-NMR (DMSO-*d*_6_): *δ* (ppm) 21.88 (CH_2_), 25.11 (CH_3_), 28.80 (CH_2_), 45.94 (CH_2_-N), 123.51, 125.62, 126.21, 127.15, 131.61, 132.54, 141.41, 154.11 (Ar-C), 184.42 (C=O); MS *m*/*z* (%): 660 (M^+^ +4, 4), 658 (M^+^ +2, 14), 656 (M^+^, 35). Anal. Calcd. for C_26_H_24_Cl_4_N_6_O_2_S_2_ (656.02): C, 47.43; H, 3.67; N, 12.76; S, 9.74. Found C, 47.37; H, 3.54; N, 12.66; S, 9.61%.

**Diethyl 5,5′-[hexane-1,6-diyl-bis(azaneylylidene)]-bis[4-(o-tolyl)-4,5-dihydro-1,3,4-thiadiazole-2-carboxylate] (5e).**

Yellow crystals (72%); m.p. 192–194 °C; IR: *v* 3028, 2927 (C-H), 1728 (C=O), 1612 (C=N) cm^−1^; ^1^H-NMR (DMSO-*d*_6_): *δ* 1.19–1.20 (t, 6H, 2CH_2_CH_3_), 1.23–1.35 (m, 4H, -CH_2_CH_2_CH_2_CH_2_CH_2_CH_2_-), 1.55–1.58 (m, 4H, -CH_2_CH_2_CH_2_CH_2_CH_2_CH_2_-), 2.34 (s, 6H, 2Ar-CH_3_), 3.45–3.47 (m, 4H, -CH_2_CH_2_CH_2_CH_2_CH_2_CH_2_-), 4.32–4.39 (q, 4H, 2CH_2_CH_3_), 6.97–7.42 (m, 8H, Ar-H); ^13^C-NMR (DMSO-*d*_6_): *δ* (ppm) 14.84 (CH_3_-CH_2_-O), 21.19 (CH_2_), 23.11 (Ar-CH_3_), 26.77 (CH_2_), 46.72 (CH_2_-N), 56.17 (CH_3_-CH_2_-O), 120.51, 123.55, 126.74, 129.42, 132.11, 133.54, 142.84, 154.17 (Ar-C), 171.81 (C=O); MS *m*/*z* (%): 608 (M^+^, 19). Anal. Calcd. for C_30_H_36_N_6_O_4_S_2_ (608.22): C, 59.19; H, 5.96; N, 13.81; S, 10.53. Found C, 59.13; H, 5.79; N, 13.74; S, 10.68%.

**Diethyl 5,5′-[hexane-1,6-diyl-bis(azaneylylidene)]-bis[4-(4-chlorophenyl)-4,5-dihydro- 1,3,4-thiadiazole-2-carboxylate] (5f).**

Yellowish brown crystals (76%); m.p. 177–179 °C; IR (KBr): *v* 3039, 2927 (CH), 1731 (C=O), 1601 (C=N) cm^−1^; ^1^H-NMR (DMSO-*d*_6_): *δ* 1.18–1.21 (t, 6H, 2CH_2_CH_3_), 1.24–1.33 (m, 4H, -CH_2_CH_2_CH_2_CH_2_CH_2_CH_2_-), 1.55–1.67 (m, 4H, -CH_2_CH_2_CH_2_CH_2_CH_2_CH_2_-), 3.45–3.47 (m, 4H, -CH_2_CH_2_CH_2_CH_2_CH_2_CH_2_-)*,* 4.32-4.39 (q, 4H, 2CH_2_CH_3_), 6.99–7.44 (m, 8H, Ar-H); ^13^C-NMR (DMSO-*d*_6_): *δ* (ppm) 14.88 (CH_3_-CH_2_-O), 21.17 (CH_2_), 26.77 (CH_2_), 46.76 (CH_2_-N), 56.52 (CH_3_-CH_2_-O), 121.51, 126.74, 129.11, 135.54, 146.84, 155.17 (Ar-C), 170.11 (C=O); MS *m*/*z* (%): 478 (M^+^ +2, 11), 650 (M^+^ +2, 6), 648 (M^+^, 14). Anal. Calcd. for C_28_H_30_Cl_2_N_6_O_4_S_2_ (648.11): C, 51.77; H, 4.66; N, 12.94; S, 9.87. Found C, 51.59; H, 4.72; N, 13.02; S, 10.05%.

**Diethyl 5,5′-[hexane-1,6-diyl-bis(azaneylylidene)]-bis[4-(2,4-dichlorophenyl)-4,5- dihydro-1,3,4-thiadiazole-2-carboxylate] (5g).**

Brown solid (78%); m.p. 192–194 °C; IR (KBr): *v* 3064, 2927 (CH), 1728 (C=O), 1603 (C=N) cm^−1^; ^1^H-NMR (DMSO-*d*_6_): *δ* 1.18–1.23 (t, 6H, 2CH_2_CH_3_), 1.28–1.36 (m, 4H, -CH_2_CH_2_CH_2_CH_2_CH_2_CH_2_-), 1.55–1.64 (m, 4H, -CH_2_CH_2_CH_2_CH_2_CH_2_CH_2_-), 3.43–3.46 (m, 4H, -CH_2_CH_2_CH_2_CH_2_CH_2_CH_2_-)*,* 4.37–4.39 (q, 4H, 2CH_2_CH_3_), 6.86–7.81 (m, 6H, Ar-H); ^13^C-NMR (DMSO-*d*_6_): *δ* (ppm) 14.11 (CH_3_-CH_2_-O), 21.19 (CH_2_), 26.68 (CH_2_), 46.68 (CH_2_-N), 56.64 (CH_3_-CH_2_-O), 123.51, 125.74, 126.11, 131.54, 133.58, 141.84, 148.17, 154.17 (Ar-C), 170.13 (C=O); MS *m*/*z* (%): 720 (M^+^ +4, 4), 718 (M^+^ +2, 14), 716 (M^+^, 35). Anal. Calcd. for C_28_H_28_Cl_4_N_6_O_4_S_2_ (716.04): C, 46.81; H, 3.93; N, 11.70; S, 8.92. Found C, 46.71; H, 3.80; N, 11.57; S, 8.79%.

**5,5′-[Hexane-1,6-diyl-bis(azaneylylidene)]bis[*N*,4-diphenyl-4,5-dihydro-1,3,4-thiadiazole-2-carboxamide] (5h).**

Yellow solid (75%); m.p. 188–190 °C; IR (KBr): *v* 3271 (NH), 3025, 2924 (C-H), 1644 (C=O), 1602 (C=N) cm^−1^; ^1^H-NMR (DMSO-*d*_6_): *δ* (ppm) 1.03–1.05 (m, 4H, -CH_2_CH_2_CH_2_CH_2_CH_2_CH_2_-), 1.19–1.23 (m, 4H, -CH_2_CH_2_CH_2_ CH_2_CH_2_CH_2_-), 3.37–3.42 (m, 4H, -CH_2_CH_2_CH_2_CH_2_CH_2_CH_2_-), 6.97–7.54 (m, 20H, Ar-H), 10.42 (s, 2H, 2NH); ^13^C-NMR (DMSO-*d*_6_): *δ* (ppm) 21.08 (CH_2_), 25.81 (CH_2_), 44.51 (CH_2_-N), 121.51, 123.62, 126.21, 127.71, 128.15, 131.55, 133.57, 137.64, 146.61, 155.11 (Ar-C), 161.42 (C=O); MS *m*/*z* (%): 674 (M^+^, 38). Anal. Calcd. for C_36_H_34_N_8_O_2_S_2_ (674.22): C, 64.07; H, 5.08; N, 16.60; S, 9.50. Found C, 64.12; H, 5.03; N, 16.42; S, 9.39%.

***N*,*N*′-(Hexane-1,6-diyl)bis[3,4-diphenylthiazol-2(3*H*)-imine] (8a).**

Yellow solid (76%); m.p. 171–173 °C; IR (KBr): *v* 2924 (C-H), 1578 (C=N) cm^−1^; ^1^H-NMR (DMSO-*d*_6_): *δ* 1.02–1.05 (m, 4H, -CH_2_CH_2_CH_2_CH_2_CH_2_CH_2_-), 1.19–1.23 (m, 4H, -CH_2_CH_2_CH_2_ CH_2_CH_2_CH_2_-), 3.43–3.55 (m, 4H, -CH_2_CH_2_CH_2_CH_2_CH_2_CH_2_-), 6.99–7.95 (m, 22H, Ar-H & thiazole-H); ^13^C-NMR (DMSO-*d*_6_): *δ* (ppm) 21.11 (CH_2_), 24.81 (CH_2_), 46.31 (CH_2_-N), 122.51, 124.62, 125.21, 127.71, 128.15, 128.94, 129.55, 131.27, 134.57, 137.64, 158.11 (Ar-C); MS *m*/*z* (%): 586 (M^+^, 18). Anal. Calcd. for C_36_H_34_N_4_S_2_ (586.22): C, 73.69; H, 5.84; N, 9.55; S, 10.93. Found C, 73.58; H, 5.69; N, 9.38; S, 11.06%.

***N*,*N*′-(Hexane-1,6-diyl)bis[3-phenyl-4-(p-tolyl)thiazol-2(3*H*)-imine] (8b).**

Yellow solid (79%); m.p. 169–171 °C; IR (KBr): *v* 2934 (C-H), 1599 (C=N) cm^−1^; ^1^H-NMR (DMSO-*d*_6_): *δ* 1.02–1.06 (m, 4H, -CH_2_CH_2_CH_2_CH_2_CH_2_CH_2_-), 1.19–1.38 (m, 4H, -CH_2_CH_2_CH_2_ CH_2_CH_2_CH_2_-), 2.35 (s, 6H, 2Ar-CH_3_), 3.34–3.51 (m, 4H, -CH_2_CH_2_CH_2_CH_2_CH_2_CH_2_-), 6.95-7.84 (m, 20H, Ar-H & thiazole-H); ^13^C-NMR (DMSO-*d*_6_): *δ* (ppm) 21.19 (CH_2_), 23.31 (CH_3_), 26.58 (CH_2_), 46.11 (CH_2_-N), 122.42, 123.58, 124.11, 125.51, 126.34, 127.52, 128.11, 129.74, 130.95, 135.84, 148.17 (Ar-C); MS *m*/*z* (%): 614 (M^+^, 22). Anal. Calcd. for C_38_H_38_N_4_S_2_ (614.25): C, 74.23; H, 6.23; N, 9.11; S, 10.43. Found C, 74.14; H, 6.19; N, 9.00; S, 10.51%.

***N*,*N*′-(Hexane-1,6-diyl)bis[4-(4-chlorophenyl)-3-phenylthiazol-2(3*H*)-imine] (8c).**

Yellow solid (82%); m.p. 201–203 °C; IR (KBr): *v* 2931 (C-H), 1602 (C=N) cm^−1^; ^1^H-NMR (DMSO-*d*_6_): *δ* 1.02–1.05 (m, 4H, -CH_2_CH_2_CH_2_CH_2_CH_2_CH_2_-), 1.42–1.51 (m, 4H, -CH_2_CH_2_CH_2_ CH_2_CH_2_CH_2_-), 3.29–3.32 (m, 4H, -CH_2_CH_2_CH_2_CH_2_CH_2_CH_2_-), 6.88–7.64 (m, 20H, Ar-H & thiazole-H); ^13^C-NMR (DMSO-*d*_6_): *δ* (ppm) 21.15 (CH_2_), 26.73 (CH_2_), 46.08 (CH_2_-N), 122.93, 123.45, 124.11, 125.42, 126.51, 127.11, 128.46, 129.13, 133.11, 146.65, 154.18 (Ar-C); MS *m*/*z* (%): 656 (M^+^ +2, 8), 654 (M^+^, 25). Anal. Calcd. for C_36_H_32_Cl_2_N_4_S_2_ (654.14): C, 65.94; H, 4.92; N, 8.54; S, 9.78. Found C, 65.83; H, 4.80; N, 8.39; S, 9.64%.

***N*,*N*′-(Hexane-1,6-diyl)bis[4-(4-bromophenyl)-3-phenylthiazol-2(3*H*)-imine] (8d).**

Yellow solid (79%); m.p. 211–213 °C; IR (KBr): *v* 2931 (C-H), 1602 (C=N) cm^−1^; ^1^H-NMR (DMSO-*d*_6_): *δ* 1.04–1.07 (m, 4H, -CH_2_CH_2_CH_2_CH_2_CH_2_CH_2_-), 1.32–1.41 (m, 4H, -CH_2_CH_2_CH_2_ CH_2_CH_2_CH_2_-), 3.41–3.67 (m, 4H, -CH_2_CH_2_CH_2_CH_2_CH_2_CH_2_-), 6.85–7.65 (m, 20H, Ar-H & thiazole-H); ^13^C-NMR (DMSO-*d*_6_): *δ* (ppm) 21.11 (CH_2_), 26.71 (CH_2_), 45.88 (CH_2_-N), 122.91, 123.35, 124.09, 125.12, 126.38, 127.11, 128.16, 129.13, 132.89, 145.63, 154.12 (Ar-C); MS *m*/*z* (%): 744 (M^+^ +2, 10), 742 (M^+^, 26). Anal. Calcd. for C_36_H_32_Br_2_N_4_S_2_ (742.04): C, 58.07; H, 4.33; N, 7.52; S, 8.61. Found C, 58.19; H, 4.24; N, 7.39; S, 8.72%.

***N*,*N*′-(Hexane-1,6-diyl)bis[4-(4-nitrophenyl)-3-phenylthiazol-2(3*H*)-imine] (8e).**

Yellow solid (77%); m.p. 193–195 °C; IR (KBr): *v* 2928 (C-H), 1602 (C=N) cm^−1^; ^1^H-NMR (DMSO-*d*_6_): *δ* 1.04–1.08 (m, 4H, -CH_2_CH_2_CH_2_CH_2_CH_2_CH_2_-), 1.36–1.52 (m, 4H, -CH_2_CH_2_CH_2_ CH_2_CH_2_CH_2_-), 3.36–3.42 (m, 4H, -CH_2_CH_2_CH_2_CH_2_CH_2_CH_2_-), 6.88–8.34 (m, 20H, Ar-H & thiazole-H); ^13^C-NMR (DMSO-*d*_6_): *δ* (ppm) 21.54 (CH_2_), 26.92 (CH_2_), 46.18 (CH_2_-N), 122.92, 123.39, 125.09, 126.12, 127.38, 128.11, 129.16, 134.13, 145.89, 147.61, 158.12 (Ar-C); MS *m*/*z* (%): 676 (M^+^, 38). Anal. Calcd. for C_36_H_32_N_6_O_4_S_2_ (676.19): C, 63.89; H, 4.77; N, 12.42; S, 9.47. Found C, 63.75; H, 4.58; N, 12.30; S, 9.57%.

**1,1′-[Hexane-1,6-diyl-bis(azaneylylidene)]bis[4-methyl-3-phenyl-2,3-dihydrothiazole-5-yl-2-ylidene]bis(ethan-1-one) (11a).**

Yellow solid (72%); m.p. 185–187 °C; IR (KBr): *v* 2930 (C-H), 1684 (C=O), 1600 (C=N) cm^−1^; ^1^H-NMR (DMSO-*d*_6_): *δ* 1.03–1.05 (m, 4H, -CH_2_CH_2_CH_2_CH_2_CH_2_CH_2_-), 1.21–1.43 (m, 4H, -CH_2_CH_2_CH_2_ CH_2_CH_2_CH_2_-), 2.28 (s, 6H, 2COCH_3_), 2.55 (s, 6H, 2 thiazole-CH_3_), 3.30–3.41 (m, 4H, -CH_2_CH_2_CH_2_CH_2_CH_2_CH_2_-), 6.93–7.35 (m, 10H, Ar-H); ^13^C-NMR (DMSO-*d*_6_): *δ* (ppm) 16.85 (thiazole-CH_3_), 21.12 (CH_2_), 24.33 (COCH_3_), 26.83 (CH_2_), 46.54 (CH_2_-N), 118.11, 122.51, 126.62, 129.21, 141.15, 144.61, 154.11 (Ar-C), 181.48 (C=O); MS *m*/*z* (%): 546 (M^+^, 20). Anal. Calcd. for C_30_H_34_N_4_O_2_S_2_ (546.21): C, 65.90; H, 6.27; N, 10.25; S, 11.73. Found C, 65.97; H, 6.09; N, 10.16; S, 11.87%.

**Diethyl2,2′-[hexane-1,6-diyl-bis(azaneylylidene)]-bis[4-methyl-3-phenyl-2,3-dihydrothiazole-5-carboxylate] (11b).**

Yellow solid (81%); m.p. 207–209 °C; IR (KBr): *v* 2927 (C-H), 1708 (C=O), 1601 (C=N) cm^−1^; ^1^H-NMR (DMSO-*d*_6_): *δ* 1.03–1.05 (m, 4H, -CH_2_CH_2_CH_2_CH_2_CH_2_CH_2_-), 1.16–1.18 (t, 6H, 2CH_2_CH_3_), 1.41–1.48 (m, 4H, -CH_2_CH_2_CH_2_CH_2_CH_2_CH_2_-), 2.57 (s, 6H, 2 thiazole-CH_3_), 3.85–3.92 (m, 4H, -CH_2_CH_2_CH_2_CH_2_CH_2_CH_2_-), 4.12–4.16 (q, 4H, 2CH_2_CH_3_), 6.89–7.34 (m, 10H, Ar-H); ^13^C-NMR (DMSO-*d*_6_): *δ* (ppm) 14.24 (CH_3_-CH_2_-O), 17.25 (thiazole-CH_3_), 21.18 (CH_2_), 26.74 (CH_2_), 46.62 (CH_2_-N), 56.95 (CH_3_-CH_2_-O), 122.51, 126.55, 128.74, 129.40, 143.11, 153.54, 158.17 (Ar-C), 170.11 (C=O); MS *m*/*z* (%): 606 (M^+^, 16). Anal. Calcd. for C_32_H_38_N_4_O_4_S_2_ (606.23): C, 63.34; H, 6.31; N, 9.23; S, 10.57. Found C, 63.25 H, 6.20; N, 9.18; S, 10.48%.

### 3.2. Molecular Docking 

Compounds are drawn using the Avogadro software 1.2.0, where the universal force field (UFF) was utilized to optimize the structures [37,38]. The docking was performed using AutoDock Vina software using the flexible ligand in the flexible active site (H41 and C145) protocol [39,40]. SARS-CoV-2 main protease M^pro^ dimer structure (PDB ID: 6Y2G) was downloaded from the protein data bank (https://www.rcsb.org/) (accessed on 20 March 2020) [27]. O6K, α-ketoamide inhibitor, is the positive control molecule that was solved in the M^pro^ structure and was used to examine the affinity of the compounds **1**, **5a**–**h**, **8a**–**e**, and **11a**,**b** to the M^pro^ active site. O6K is a covalently bound ligand, but we redock it to the solved structure in a non-bonded fashion, and it gives a root-mean-square difference of 0.966 Å. Additionally, the peptidyl Michael acceptor, N3, found in the solved structure of Mpro (PDB ID: 6LU7) is also used as a positive control for comparison [30]. The M^pro^ dimer was subjected to molecular dynamics simulation (MDS) which ran for 80 nanoseconds, as reported [41] before the docking study. The MDS was conducted on the WEBGRO macromolecular simulation utilizing GROningen MAchine for Chemical Simulations (GROMACS) software and CHARMM27 force field [42,43]. A minimization for 10,000 steps of the conjugate gradient is performed before the MDS run. TIP4P water model was used with a constant number of atoms, volume, and temperature (NVT) ensemble in cubic periodic boundary conditions. Na^+^ and Cl^−^ were added to the system for the salt concentration of 154 mM, while the temperature and pressure were adjusted to be 310 K and 1 atm, respectively, to resemble physiological conditions. Clustering of the trajectories was performed using the UCSF Chimera 1.14 software [44]. A representative structure from each cluster was used when testing the binding affinity using the AutoDock Vina software [39,45]. 

The search box was adjusted to cover the active site dyad (H41 and C145) with dimensions of 30 Å × 30 Å × 30 Å centered at (25.2, 47.3, 38.4) Å. Protein–ligand interaction profiler (PLIP) webserver (https://plip-tool.biotec.tu-dresden.de/plip-web/plip/index) (accessed on 5 May 2021) was utilized to check the binding modes and the data are tabulated and then represented using PyMOL software 2.0.4 in the results section [46,47,48]. In the current study, we used an exhaustiveness value of 100. This is due to the many rotatable bonds we have in some ligands.

## 4. Conclusions

The present study disclosed the preparation of 1,1′-(hexane-1,6-diyl)bis(3-phenylthiourea) which was employed as a key intermediate for the synthesis of a new series of *bis*-[1,3,4]thiadiazolimines, and *bis*-thiazolimines, with an alkyl linker, through its reaction with various hydrazonoyl halides or *α*-haloketones, respectively. The newly synthesized derivatives’ structures were confirmed by elemental analysis and spectral data. Docking studies were applied to test the binding affinity of the synthesized products against the M^pro^ of SARS-CoV-2. The study results showed that compound **5h** is the best one as it has average binding energy (−7.50 ± 0.58 kcal/mol) better than that of the positive controls, O6K and N3 (−7.36 ± 0.34 and −6.36 ± 0.31 kcal/mol). Additionally, the docking poses (H-bonds and hydrophobic contacts) of the tested compounds against the M^pro^ using the PLIP web server were analyzed. This work paves the way for the design and synthesis of *bis*-thiadiazoles and *bis*-thiazoles-based libraries, which could lead to the innovation of efficient treatment against SARS-CoV-2 main protease (M^pro^).

## Figures and Tables

**Table 1:** The interaction pattern of the compounds (**1**, **5a**–**h**, **8a**–**e**, and **11a**,**b**) and the positive control O6K against SARS-CoV-2 M^pro^.

Compd	Binding Energy (kcal/mol)	H-Bonding or Halogen Bonds		Hydrophobic Interaction	
		Number	Residues Take Part in the Interaction	Number	Residues Take Part in the Interaction
**O6K**	−7.4	8	S1, **H41**, **G143**, **S144**, **C145**, H164, and **E166**(**2**)	4	**N142**, M165, D187, and **Q189**
**N3**	−6.6	2	H164 and **E166**	3	T25, T26, and P168
**1**	−5.8	2	**E166** and T190		
**5a**	−6.9	4	**G143**, **S144**, **C145**, and **Q189**	3	M165, **E166**, and **Q189**
**5b**	−6.7	4	**G143**, **S144**, **C145**, and Q192	2	P39 and D187
**5c**	−6.8	2 1	**G143** and **S144** R188	1	**Q189**
**5d**	−6.9	2 1	**G143** and **S144** R188	2	**N142** and **Q189**
**5e**	−6.8	2	**N142** and **E166**	6	T25, **N142**, M165, **E166**, P168, and **Q189**
**5f**	−6.6	3	**G143**, **S144**, and **C145**	4	F140, M165, P168, and **Q189**
**5g**	−6.9	2 1	**G143** and **S144** T190	5	T25, Y118, M165, **E166**, and **Q189**
**5h**	**−7.5**			5	F140, **E166**(**2**), P168, and **Q189**
**8a**	−6.9			3	**N142**, **E166**, and L167
**8b**	−7.2			4	**N142**, **E166**, L167, and P168
**8c**	−7.0			4	**N142**, M165, **E166**, and L167
**8d**	−7.0			2	**N142** and **E166**
**8e**	−7.1			4	**N142**, **E166**, and **Q189**(**2**)
**11a**	−6.5	3	**G143**, **S144**, and **C145**	2	**Q189**(**2**)
**11b**	−6.3	3	**G143**, **S144**, and **C145**	2	**Q189**(**2**)

## Data Availability

The data presented in this study are available on request from corresponding author.
